# Genome Sequences of Two *Bacillus cereus* Group Bacteriophages, Eyuki and AvesoBmore

**DOI:** 10.1128/genomeA.01199-15

**Published:** 2015-10-15

**Authors:** Ivan Erill, Steven M. Caruso

**Affiliations:** Department of Biological Sciences, University of Maryland, Baltimore County, Baltimore, Maryland, USA

## Abstract

The genomes of two double-stranded DNA (dsDNA) bacteriophages isolated on *Bacillus thuringiensis* show similarity to previously sequenced phages and provide evidence of the mosaicism of phage genomes.

## GENOME ANNOUNCEMENT

Here, we present the genomes of the *Bacillus* bacteriophages Eyuki and AvesoBmore, which were isolated from soil samples collected from Ellicott City and Baltimore, MD, by undergraduate student researchers as part of the SEA-PHAGES program (1). Additional information about these and other *Bacillus* phages isolated by undergraduate researchers can be found at http://bacillus.phagesdb.org/. These double-stranded DNA phages were isolated on *Bacillus thuringiensis* subsp. *kurstaki* ATCC 33679, a Gram-positive, endospore-forming, and rod-shaped member of the *Bacillus cereus* group (2). Eyuki and AvesoBmore were sequenced by Illumina sequencing to 1,653× and 3,425× coverage and were determined to have genomes of 162,252 bp and 167,431 bp in length, with 38.8% and 37.7% G+C content, respectively, each having direct terminal repeats.

Examination of the genomes revealed Eyuki to contain 300 protein-coding genes and AvesoBmore to have 302 protein-coding genes, with neither phage containing tRNA genes. We applied an iterative method to identify the translational codon usage signature of bacterial genomes to *B. thuringiensis* and other bacterial species and used this pattern to analyze codon usage bias in the Eyuki and AvesoBmore genomes (3). The codon usage bias of both phages shows clear evidence of adaptation to its host and, more broadly, to the *B. cereus* group, with structural genes showing significant enrichment in translational codon bias.

Cluster C phages have several notable characteristics in terms of gene content. Of particular interest is the presence of a RecA homolog and a sporulation-like sigma F subunit of RNA polymerase. Using phylogenetic analyses, we reconstructed the evolutionary history of several phage proteins. Our results show clear evidence of lateral gene transfer from bacteria in some instances. They also support the idea that the sigma F homolog found in these phages derives from the sporulation sigma factor of the *Bacillaceae* and points to a eukaryotic/archeal origin of the RecA-like recombination protein. Combined, these results highlight the diverse genetic history of these large-genome phages and reveal evidence of substantial mosaicism. Comparisons of Eyuki and AvesoBmore with previously characterized *Bacillus* phages at the DNA and protein levels show Eyuki to be closely related to WPh (GenBank accession no. HM144387.1), Hakuna (accession no. KJ489399.1), and Megatron (accession no. KJ489401.1), which found the forthcoming C1 subcluster of *Bacillus* phages, while AvesoBmore is closely related to Troll (accession no. KF208639.2) and Riley (accession no. KJ489402.1), the soon-to-be-christened C3 phages (A. B. Sauder, M. R. Quin, A. Brouillette, S. M. Caruso, S. Cresawn, L. Lewis, K. Loesser-Caser, M. Pate, C. Scott, S. Stockwell, and L. Temple, unpublished data). An intron containing a putative homing endonuclease (4) was identified in an Eyuki gene-encoding DNA polymerase, as has been described in *Bacillus* phages CAM003, Hoody T, and Megatron (see Sauder et al.).

### Nucleotide sequence accession numbers.

The complete genome sequences of the *Bacillus* phages Eyuki, accession no. KT207918, and AvesoBmore, accession no. KT307976, are deposited in GenBank.
